# Carotid artery corrected flow time and respiratory variations of peak blood flow velocity for prediction of hypotension after induction of general anesthesia in elderly patients

**DOI:** 10.1186/s12877-022-03619-x

**Published:** 2022-11-19

**Authors:** Ji Wang, Yulan Li, Hang Su, Juan Zhao, Faping Tu

**Affiliations:** 1 Department of Anesthesiology, Affiliated Hospital of North Sichuan Medical College, No.1 Maoyuan South Road, Nanchong, 637000 Sichuan China; 2 Department of Anesthesiology, Zigong First People’s Hospital, Zigong, Sichuan China; 3 Department of Anesthesiology, School of Medicine, Sichuan Cancer Hospital and Institute, Sichuan Cancer Center, University of Electronic Science and Technology of China, Chengdu, Sichuan China

**Keywords:** Carotid Doppler ultrasound, Corrected flow time, Blood flow velocity, Respiration, Elderly, Postinduction hypotension, General anesthesia

## Abstract

**Background:**

Postinduction hypotension is closely related to postoperative complications. Elderly patients with compromised cardiovascular compensatory reserve are more susceptible to hypotension after induction of general anesthesia. This study investigated whether the carotid artery corrected flow time (FTc) and respiratory variation of peak blood flow velocity in the common carotid artery (ΔVpeak) could predict postinduction hypotension in elderly patients.

**Methods:**

This prospective observational study included elderly patients aged 65 to 75 who were scheduled for elective surgery under general anesthesia with ASA physical status class of I-II, without cardiovascular disease, hypertension, diabetes, or obesity. Anesthesia was induced by midazolam, sufentanil, and etomidate and was maintained by sevoflurane. The carotid artery FTc and ΔVpeak were measured by ultrasound before induction of anesthesia. Hemodynamic data were recorded before induction and then during the first 10 min after induction.

**Results:**

Ninety-nine patients were included in the final analysis, of whom 63 developed postinduction hypotension. The area under the receiver operating characteristic curves was 0.87 (0.78 to 0.93) for carotid artery FTc and 0.67 (0.56 to 0.76) for ΔVpeak, respectively. The optimal cutoff value for predicting postinduction hypotension was 379.1 ms for carotid artery FTc, with sensitivity and specificity of 72.2 and 93.7%, respectively. The best cutoff value was 7.5% for ΔVpeak, with sensitivity and specificity of 55.6 and 75.0%, respectively.

**Conclusions:**

The carotid artery FTc is a reliable predictor of postinduction hypotension in elderly patients with ASA status of I or II, without cardiovascular disease, hypertension, diabetes, or obesity. Elderly patients with a carotid artery FTc less than 379.1 ms before anesthesia have a higher risk of postinduction hypotension.

**Trial registration:**

Clinical Trial Registry on August 2nd, 2020 (www.chictr.org.cn; ChiCTR2000035190).

## Background

Hypotension after induction of general anesthesia, or postinduction hypotension, is quite common in clinical practice, and if severe or prolonged, may cause organ hypoperfusion and ischemia, and may increase the incidence of postoperative adverse outcomes such as myocardial injury, ischemic stroke, acute kidney injury, and even increase 1-year mortality [1–5]. After induction of anesthesia, patients are at particular risk of hypotension mainly due to preexisting hypovolemia, the cardiovascular depressant and vasodilatory effects of induction agents, and a lack of surgical stimulation [6–8]. Previous studies have revealed that older age is an independent risk factor for the development of postinduction hypotension [9–11]. Elderly patients have a high prevalence of left ventricular diastolic dysfunction, decreased vascular reactivity, and increased sensitivity to anesthetics, and thus, are prone to hemodynamic fluctuation and hypotension. More importantly, elderly patients are less able to tolerate any period of hypotension [5]. Therefore, predicting hypotension after induction of anesthesia in elderly patients is of important clinical value for early intervention and reduction of postoperative complications and mortality.

Recently, ultrasonography for assessing volume status or predicting fluid responsiveness as well as post-induction hypotension has been widely applied in different clinical scenarios [6, 12–16]. However, venous ultrasound indices, represented by inferior vena cava (IVC) diameter and IVC collapsibility index, are less reliable in predicting fluid responsiveness in spontaneous breathing patients, as they are susceptible to the patient’ respiratory effort and patterns [17, 18]. Doppler corrected flow time (FTc) refers to the left ventricular ejection time corrected by heart rate and is known to be proportional to left ventricular preload and cardiac inotropy and inversely proportional to systemic vascular resistance [19, 20]. Previous studies have found that the FTc of carotid artery is unaffected by respiration [21]. Both carotid artery FTc and respiratory variation of peak blood flow velocity in the common carotid artery (ΔVpeak) can be good predictors of fluid responsiveness in spontaneous breathing patients [14, 15]. Furthermore, Maitra et al. [16] demonstrated that the carotid artery FTc is a good predictor of postinduction hypotension in ASA status I and II adult patients, but their study did not include elderly patients.

The aim of this study, therefore, was to evaluate the predictive power of carotid artery FTc and ΔVpeak measured preoperatively by bedside ultrasound in predicting hypotension after induction of general anesthesia in elderly patients undergoing elective surgery.

## Methods

### Study design, setting and participants

This prospective observational study was conducted in the Affiliated Hospital of North Sichuan Medical College from September 2020 to December 2020. Elderly patients who were 65 to 75 years of age with an American Society of Anesthesiologists (ASA) physical status class of I-II and scheduled for elective surgery under general anesthesia were recruited in this study. Patients with a body mass index (BMI) < 18 kg/m^2^ or > 30 kg/m^2^, cardiac rhythm other than sinus, a history of hypertension, diabetes, coronary heart disease, cardiac disease including cardiomyopathy and mild to severe valve disease, pulmonary hypertension, peripheral arterial disease or atherosclerosis, preoperative cervical vascular ultrasound abnormalities including plaque, stenosis and anatomic variation, and any previous neck surgery or trauma were excluded. This study was approved by the Ethics Committee of the Affiliated Hospital of North Sichuan Medical College, Nanchong, China (2020ER082-1) and registered in the Chinese Clinical Trial Registry in August 2nd, 2020 (www.chictr.org.cn; ChiCTR2000035190). Written informed consent was obtained from all participants.

### Study procedure

All patients underwent routine fasting for at least 6 to 8 h and were not allowed to drink any solution or fluid 2 to 4 h prior to surgery. After entering the operating room, the patients were placed in a supine position. The Bene View N15 monitor (Mindray Biomedical Electronics Co., Shenzhen, China) was attached to monitor the electrocardiography, pulse oximetry, heart rate (HR), noninvasive blood pressure (BP), including systolic blood pressure, diastolic blood pressure, and mean arterial pressure (MAP). The bispectral index (BIS; Aspect Medical Systems, Inc., USA) was connected to evaluate the anesthetic depth. Meanwhile, intravenous access was established and crystalloid fluid was infused at a rate of 10 ml/kg/h. Then the patients were kept quietly for at least 10 min before the carotid artery ultrasonography. After the measurement of carotid artery FTc and ΔVpeak, HR and BP were recorded for 3 min at 1-min intervals, and the mean values of HR and MAP were taken as the baseline value. Finally, anesthesia induction and endotracheal intubation were sequentially executed, and the HR and BP were measured every minute for 10 min after the induction of anesthesia. The lowest value of HR and MAP during this period was used to calculate the percentage decrease in HR and MAP of each patient separately: percentage decrease in HR = (baseline HR- lowest HR)/baseline HR × 100%; percentage decrease in MAP = (baseline MAP- lowest MAP)/baseline MAP × 100%. The BIS value at the moment of lowest MAP was recorded.

### Carotid artery ultrasonography

**Fig. 1:**
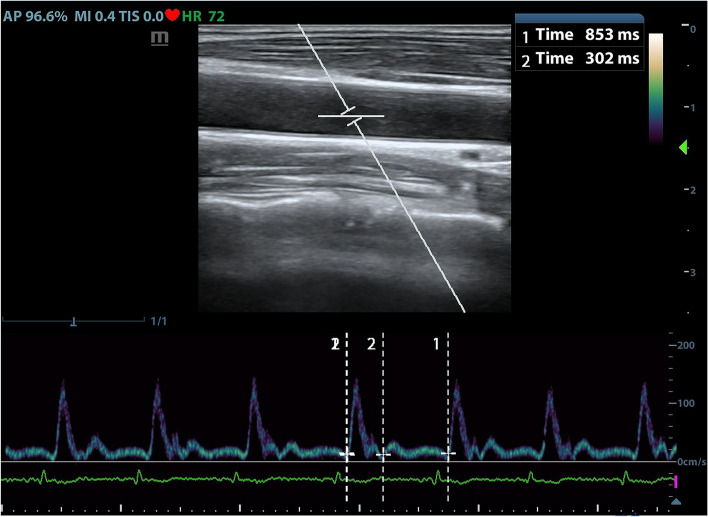
Ultrasound measurements of the carotid artery and calculation of FTc. The panel above shows a two-dimensional scan of the right common carotid artery, and the following panel shows the corresponding blood flow spectrum. “1 Time” indicates the systolic flow time (ST), and “2 Time” presents the cycle time (CT)

**Fig. 2:**
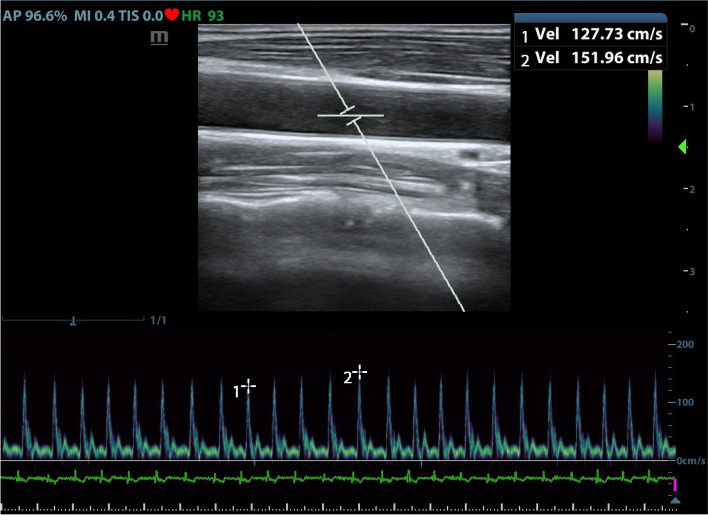
Ultrasound measurements of the carotid artery and calculation of ΔVpeak. The panel above shows a two-dimensional scan of the right common carotid artery, and the following panel shows the corresponding blood flow spectrum. “1 vel” and “2vel” represent the minimum and maximum values of peak blood flow velocity, respectively, in a single respiratory cycle

Ultrasound measurements were performed under a vascular setting with a 4–12 MHz linear transducer and ultrasound device (M9Sc-D system; Mindray Inc., China). Carotid artery FTc and ΔVpeak measurements were performed by one trained examiner who was excluded from the study design. The ultrasonic data extraction was conducted by another examiner who was unaware of the patient’s hemodynamic parameters. The right common carotid artery FTc and ΔVpeak were measured in patients with their heads tilted 30° to the left, as previously described by Blehar et al. [22] and Song et al. [23]. Briefly, a long axis view of the right common carotid artery was obtained at the lower border of the thyroid cartilage, and color flow was placed over the blood vessel approximately 2 cm proximal to the carotid artery bifurcation. Then, pulse wave Doppler was selected, and the sampling frame was placed in the area where the carotid artery had its best color flow, with an angle of less than 60°, to obtain the blood flow spectrum. After that, using the caliper function of the machine, cycle time (CT) was measured from the beginning of the systole to the beginning of the following systole, and systolic flow time (ST) was measured from the beginning of the systolic upstroke to the dicrotic notch (Fig. 1). The FTc of the carotid artery was calculated using Bazett’s formula, FTc = ST/$\surd CT$ [24]. Three carotid artery FTc values were generated from three consecutive cardiac cycles, and the mean of these values was used for analysis. ΔVpeak was measured by tracking the blood flow spectrum with a slowed scanning speed under the same measurement condition as FTc, where the maximum and minimum peak systolic velocities were obtained in a single respiratory cycle, as shown in Fig. 2. The ΔVpeak was calculated as follows: ΔVpeak = (maximum peak velocity – minimum peak velocity) / [(maximum peak velocity + minimum peak velocity)/2] × 100%. Three ΔVpeak values were obtained from three consecutive respiratory cycles, and the average of these values was used for analysis.

### Anesthesia protocol

No premedication was given before induction. Anesthesia induction was performed with 0.04 mg/kg midazolam, 0.4 µg/kg sufentanil, and 0.3 mg/kg etomidate, while endotracheal intubation was facilitated with intravenous 0.6 mg/kg rocuronium. After 3 min of mask ventilation, tracheal intubation was performed by an attending anesthesiologist who was blinded to the ultrasound data. There were no episodes of desaturation or difficult intubation. Anesthesia was maintained with inhaled 1–2% sevoflurane to reach a target BIS value of 40–60. Patients were volume-controlled ventilated (tidal volume: 8 ml/kg; respiratory rate: 10–12 breath/min) to maintain an end-tidal carbon dioxide partial pressure between 35 and 40 mmHg. Postinduction hypotension was defined as an absolute MAP of less than 65 mmHg or a more than 20% drop in MAP from the baseline during the recorded period after induction of anesthesia [3]. Postinduction hypotension was treated with intravenous ephedrine in 3 mg bolus doses and repeated when necessary. Significant bradycardia (heart rate < 40 beats/min) was treated with intravenous boluses of atropine (0.3 mg).

### Statistical analysis

A pilot study involving 25 subjects revealed that the incidence of postinduction hypotension in elderly patients was 68.0%. Maitra et al. [16] reported that the area under the receiver operating characteristic (ROC) curve was 0.91 for carotid artery FTc to predict postinduction hypotension in adult patients. We assumed that the carotid artery FTc might have a lower predictive validity of 0.70 for postinduction hypotension in elderly patients. As a result, the sample size calculation showed that at least 90 patients were necessary to detect a difference of 0.20 between the ROC curve of the carotid artery FTc (0.70) and the null hypothesis (0.50) [25, 26], with a power of 0.90 and a two-tailed type I error of 0.05. To allow for a possible 20% dropout rate, 108 patients were enrolled.

Normality of the data distribution was assessed with Kolmogorov–Smirnov test. Continuous variables were presented as mean ± standard deviation if normally distributed, or as medians [interquartile ranges] if not. Categorical variables were expressed as absolute numbers (%). Hypotension and non-hypotension groups were compared with an independent t-test for normally distributed data, Mann–Whitney U-test for non-normally distributed data, and χ^2^ -test or Fisher’s exact test, as appropriate, for categorical variables. The ROC curve was depicted to assess the predictability of carotid artery FTc and ΔVpeak on postinduction hypotension. The best cutoff value was chosen to maximize the Youden index. Multivariate logistic regression analysis was performed to assess the impact of age, BMI, sex, ASA physical status, baseline MAP, carotid artery FTc and ΔVpeak on the occurrence of hypotension after checking for multicollinearity. All statistical analyses were conducted using SPSS (version 25; SPSS Inc., USA) and MedCalc (version 19.6.1; MedCalc, Belgium). Statistical significance was set at *P* < 0.05.

## Results

**Fig. 3:**
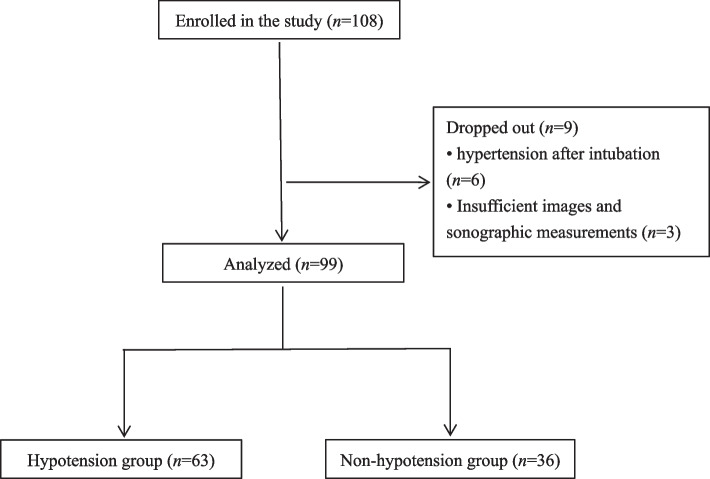
Study flow chart

Of the 108 participants assessed for eligibility, 9 were excluded because of being treated with propofol for hypertension after intubation (*n* = 6), ineffective images and sonographic measurements (*n* = 3). Thus, 99 patients completed the study without missing data (Fig. 3). Among them, 4 patients had a history of tuberculosis, 3 had a history of bronchiectasis, 3 had a history of rheumatoid arthritis, and 1 had a history of mild anemia. After induction of anesthesia, hypotension occurred in 63 cases (63.6%), of which 20.67% received a single intravenous injection of ephedrine (3 mg), and 79.37% were treated with two intravenous injections of ephedrine (total dose of 6 mg).

As shown in Table 1, there was no significant difference in age, sex, BMI, percentage of smokers, ASA physical status, baseline MAP, and baseline HR between the hypotension and non-hypotension groups. The type of surgery (thoracic, general, gynecological, otorhinolaryngologic, or orthopedic surgery), the amount of anesthesia drugs (midazolam, sufentanil, or etomidate), and the volume of infusion prior to induction of anesthesia showed no difference between the two groups as well. After induction of anesthesia, the lowest MAP in the hypotension group was significantly lower than that in the non-hypotension group (*P* < 0.001), and the percentage decrease in MAP was higher than that in the non-hypotension group (*P* < 0.001), whereas the BIS value at the moment of lowest MAP, the lowest HR, and the percentage decrease in HR were similar in the two groups. Patients who developed hypotension had a shorter carotid artery FTc (355.5 ± 22.9 vs. 386.3 ± 19.0 ms; *P* < 0.001) and a higher ΔVpeak (8.3 ± 3.8% vs. 6.1 ± 2.4%; *P* = 0.001) before anesthesia.

**Table 1:** Baseline demographic, types of surgery, anesthetic details, hemodynamic data, and preoperative carotid artery ultrasound measurements between the hypotension and non-hypotension groups

Parameter	Hypotension (*n* = 63)	Non-hypotension (*n* = 36)	*P*
Age (years)	69 [66 to 71]	68 [66 to 71]	0.842
Male/female	42/21	23/13	0.828
BMI (kg/m^2^)	22.1 ± 2.3	23.0 ± 3.2	0.141
Smoker (%)	26 (41.3%)	11 (30.6%)	0.289
ASA physical status, I/II	54/9	34/2	0.319
Thoracic surgery	21 (33.3%)	7 (19.4%)	0.168
General surgery	38 (60.3%)	25 (69.4%)	0.394
Gynecological surgery	2 (3.2%)	1 (2.8%)	0.912
Otolaryngologic surgery	1 (1.6%)	1 (2.8%)	0.685
Orthopedic surgery	1 (1.6%)	2 (5.6%)	0.299
Fluid volume before induction (ml)	234 ± 33	243 ± 33	0.216
Midazolam (mg)	2.3 ± 0.3	2.3 ± 0.3	0.215
Sufentanil (ug)	22.5 ± 3.1	23.3 ± 3.1	0.215
Etomidate (mg)	16.9 ± 2.4	17.5 ± 2.3	0.215
Baseline MAP (mmHg)	94 ± 10	92 ± 10	0.534
Lowest MAP (mmHg)	64 ± 7	77 ± 8	< 0.001
Decrease in MAP (%)	30.90 ± 6.5	16.6 ± 3.8	< 0.001
Baseline HR (bpm)	74 ± 7	76 ± 10	0.437
Lowest HR (bpm)	62 ± 7	63 ± 7	0.494
Decrease in HR (%)	16.7 ± 9.0	15.9 ± 12.9	0.693
BIS (at lowest MAP)	53 ± 2	52 ± 3	0.125
Carotid artery FTc (ms)	355.5 ± 22.9	386.3 ± 19.0	< 0.001
ΔVpeak (%)	7.8[5.5 to 10.6]	5.9[4.4 to 8.0]	0.006

Data are presented as the mean ± standard deviation, median [interquartile range] or number of patients (%)

**Fig. 4:**
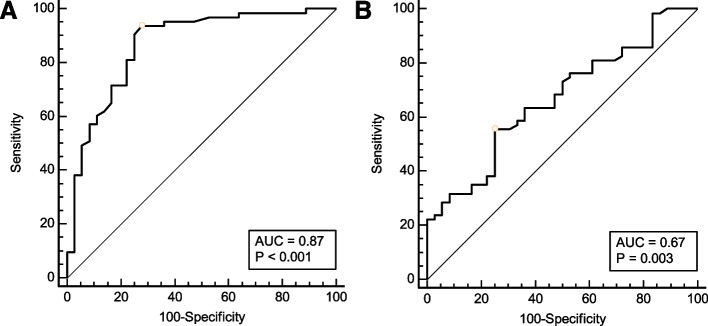
Receiver operating characteristic curves showing the predictability of carotid artery FTc (**A**) and ΔVpeak (**B**) on hypotension after induction of anesthesia in elderly patients. The circles on the curves indicate the optimal cutoff values determined by maximizing the Youden index. AUC, area under the receiver operating characteristic curve

The power of carotid artery FTc and ΔVpeak to predict postinduction hypotension is shown in Fig. 4 and Table 2. The area under the ROC curve for carotid artery FTc was 0.87 (95% CI, 0.78 to 0.93; *P* < 0.001), and the optimal cutoff value was 379.1 ms, with a sensitivity of 72.2% (95% CI, 54.8 to 85.8%) and specificity of 93.7% (95% CI, 84.5 to 98.2%). The gray zone for carotid artery FTc was 362.3–378.1 ms and included 25.3% of the patients. On the contrary, the area under the ROC curve for ΔVpeak was 0.67 (95% CI, 0.56 to 0.76; *P* = 0.003). The optimal cutoff value of ΔVpeak was 7.5%, with a sensitivity of 55.6% (95% CI, 42.5 to 68.1%) and specificity of 75.0% (95% CI, 57.8 to 87.9%), and the gray zone for ΔVpeak was 3.7–9.1% and included 62.6% of the patients.

**Table 2:** Prediction of hypotension after induction of anesthesia by receiver operating characteristic curves of the carotid artery FTc and ΔVpeak

Parameter	Area under the ROC curve (95% CI)	*P*	Optimal cutoff value	Gray zone	Patients in gray zone (%)	Sensitivity (%)(95% CI)	Specificity (%)(95% CI)	Youden Index J
Carotid artery FTc (ms)	0.87(0.78 to 0.93)	< 0.001	379.1	362.3 to 378.1	25(25.3)	72.2 (54.8 to 85.8)	93.7(84.5 to 98.2)	0.659
ΔVpeak (%)	0.67(0.56 to 0.76)	0.003	7.5	3.7 to 9.1	62 (62.6)	55.6 (42.5 to 68.1)	75.0(57.8 to 87.9)	0.306

*ROC* Receiver operating characteristic

As indicated in Table 3, multivariate logistic regression analysis identified that carotid artery FTc (Odds ratio = 0.92; 95% CI, 0.89 to 0.96; *P* < 0.001), but not ΔVpeak (*P* = 0.159), was the only independent predictor of hypotension after induction of general anesthesia in the elderly. That is, among elderly paitents, every ms decrease in carotid artery FTc increases the risk of postinduction hypotension by 8%.

**Table 3:** Results of logistic regression analysis to predict the occurrence of hypotension after induction of anesthesia

Predictors	Regression Coefficient	Odds Ratio (95% CI)	*P*
Constant	29.9	12 + e^9.908^	0.002
Age	0.048	1.05 (0.88 to 1.25)	0.588
BMI	-0.174	0.84 (0.68 to 1.04)	0.110
Female	-1.036	0.85 (0.26 to 2.77)	0.787
ASA classification			0.286
ASA I	1.0	1.0	
ASA II	-0.163	0.36 (0.05 to 2.38)	
Baseline MAP	0.013	1.01 (0.95 to 1.08)	0.668
Carotid artery FTc	-0.80	0.92 (0.89 to 0.96)	< 0.001
ΔVpeak	0.136	1.15 (0.95 to 1.39)	0.159

## Discussion

In this study, we found that preoperative carotid ultrasound measurements were predictive of hypotension after induction of anesthesia in ASA I-II elderly patients undergoing elective surgery who did not have cardiovascular disease, hypertension, diabetes, or obesity. The carotid artery FTc, but not ΔVpeak, showed a good predictive ability. The cutoff value of carotid artery FTc was 379.1 ms, with a sensitivity of 72.2%, specificity of 93.7%, and gray zones between 362.3 and 378.1 ms including 25.3% of patients.

In our study, we chose an absolute MAP less than 65 mmHg or a decrease in MAP more than 20% from baseline as the definition of hypotension, which has been demonstrated to be associated with both progressive kidney and myocardial injury [3]. Hemodynamic changes were recorded during the first 10 min after induction, during which no surgery or intense external stimulation was exerted. This time period is also the common waiting time from induction to the start of surgery at most medical centers [6, 27, 28]. In order to detect the predictive effect of carotid artery FTc and ΔVpeak on postinduction hypotension in the elderly, some previously reported risk factors including ASA III-V, a history of hypertension or diabetes, emergency surgery, use of propofol for induction, and high dosage of fentanyl were excluded [9–11]. Since preoperative hypovolemia is a major cause of hypotension after induction of general anesthesia [6], fluid infusion was started once the patients entered the operating room, and there was no difference in the amount of infusion between the hypotension and non-hypotension groups. Besides, the BIS values of the hypotension group at the lowest time of MAP were 53 ± 2, suggesting that these elderly patients were not likely to be under deep anesthesia at that moment. In this case, postinduction hypotension occurred in approximately 63.6% of the elderly patients, presumably caused by an underlying preoperative hypovolemic state and the synergistic inhibitory effect of anesthetics like midazolam [29] and etomidate [30] on the cardiovascular system.

Since the carotid artery is a superficial blood vessel located in the neck, ultrasound carotid measurements are technically easy for anesthesiologists to obtain. Furthermore, based on the rationale that both carotid artery FTc and ΔVpeak can predict fluid responsiveness in spontaneously breathing patients [14, 15] and that preoperative hypovolemia contributes to postinduction hypotension [6], carotid ultrasound measurements can serve as a useful tool for anesthesiologists to predict postinduction hypotension. Carotid artery FTc and ΔVpeak were first confirmed by Maitra and colleagues [16] to be able to predict the occurrence of hypotension after induction of general anesthesia in surgical patients. Maitra et al. [16] reported that the carotid artery FTc predicted postinduction hypotension with an area under the ROC curve of 0.91 and the best cutoff value of 330.2 ms in ASA I or II adult patients (18—65 years) undergoing elective surgery. In our current study, a similar area under the ROC curve of carotid artery FTc (0.87) was achieved, but the cutoff value (379.1 ms) was longer than that of Maitra et al. That is, elderly patient aged 65–75 years with a carotid artery FTc less than 379.1 ms at rest is more likely to develop hypotension after the induction of general anesthesia. The reason why the optimal cutoff value of carotid artery FTc in elderly patients in our study was longer than that of adult patients in Maitra et al.’s study is probably that a slower heart rate of elderly people leads to a relatively prolonged left ventricular systolic duration, which is consistent with the findings that aortic FTc is positively correlated with age [31]. On the other hand, approximately 68% of patients in Maitra et al.’s trial had hypertension, which might increase left ventricular afterload and thus shorten their carotid artery FTc [19].

Consistent with Maitra et al., we revealed a poor predictive role of ΔVpeak on postinduction hypotension in elderly patients, due to its low area under the ROC curve (only 0.67), large gray zone (including 62% of patients), and failing to achieve statistical significance in multivariate logistic regression analysis. As a dynamic index that changes with respiration, the predictive effect of ΔVpeak on postinduction hypotension is mainly based on the cardiopulmonary interaction. We can speculate that either the irregular changes in tidal volume between breaths or the insufficient intrathoracic pressure alteration to trigger an effective cardiopulmonary interaction could weaken the predictive power of ΔVpeak.

The current study has some limitations. First, we included ASA status I or II elderly patients with undetectable carotid artery plaque or stenosis under ultrasound; therefore, our findings cannot be extrapolated in patients with higher ASA grades or patients with hypertension, diabetes, obesity, and cardiac disease. Second, the induction drug regimen in our study includes midazolam, sufentanil, and etomidate. Our findings may not be applicable to elderly patients who received propofol for induction, becasue propofol may trigger a higher incidence of postinduction hypotension [32]. Third, this study was a single-center small sample study. Fourth, patients in our study did not receive any breathing training, which could lead to underestimating of the actual predictive power of ΔVpeak.

## Conclusions

In conclusion, the carotid artery FTc, but not ΔVpeak, is a reliable predictor of postinduction hypotension (induced by midazolam, sufentanil, and etomidate and maintained by sevoflurane) in ASA I or II elderly patients without metabolic or cardiovascular diseases. Elderly patients were likely to develop postinduction hypotension when the carotid artery FTc prior to anesthesia was less than 379.1 ms.

## Data Availability

The datasets used and/or analyzed in the current study are available from the corresponding author on reasonable request.

## Abbreviations

ASA: American Society of Anesthesiologist
BIS: Bispectral index
BMI: Body Mass Index
CI: Confidence interval
CT: Cycle time
FTc: Corrected flow time
IVC: Inferior vena cava
MAP: Mean arterial pressure
ROC: Receiver operating characteristic
ST: Systolic flow time
ΔVpeak: Respiratory variation of peak blood flow velocity in the common carotid artery
